# Impact of Admission Ward on Long-Term Outcomes in Patients with Non-ST Elevation Myocardial Infarction

**DOI:** 10.3390/jcm14041284

**Published:** 2025-02-15

**Authors:** Carmi Bartal, Ranin Hilu, Hadel Alsana, Ido Peles, Gal Tsaban, Miri Merkin, Gabriel Rosenstein, Aref El-Nasasra, Hezzy Shmueli, Yigal Abramowitz, Carlos Cafri, Doron Zagher, Edward Koifman

**Affiliations:** 1 Internal Medicine E Department, Soroka University Medical Center, Be’er Sheva 84101, Israel; carmibtl@gmail.com (C.B.); hadilals@gmail.com (H.A.); 2 Faculty of Health Sciences, Ben-Gurion University of the Negev, Be’er Sheva 84105, Israel; idopeles17@gmail.com (I.P.); gtsaban@gmail.com (G.T.); miriamma2@clalit.org.il (M.M.); gabrielr@bgu.ac.il (G.R.); arefsalam@hotmail.com (A.E.-N.); hezhez56@gmail.com (H.S.); yigalab@yahoo.com (Y.A.); drcarloscafri@gmail.com (C.C.); dzahger@bgu.ac.il (D.Z.); 3 Meir Medical Center, Kfar Saba 44281, Israel; eddiekoman@gmail.com; 4 Faculty of Medicine, Tel Aviv University, Tel Aviv 69978, Israel; 5 Clinical Research Center, Soroka University Medical Center and the Faculty of Health Sciences, Be’er Sheva 84101, Israel; 6 Cardiology Department, Soroka University Medical Center, Be’er Sheva 84101, Israel; 7 Mayo Clinic, Rochester, MN 55905, USA

**Keywords:** non-ST elevation myocardial infarction, hospital admission, mortality, major adverse cardiovascular events

## Abstract

**Background**: Patients presenting with non-ST elevation myocardial infarction (NSTEMI) are often admitted to medical wards. We aimed to evaluate the impact of the admitting department on long-term outcomes. **Methods**: Patients admitted to a large tertiary center were categorized according to the admission ward, either the intensive cardiac care unit (ICCU) or internal medicine department (IMD). We compared major adverse cardiovascular events (MACEs), a composite of all-cause death, recurrent myocardial infarction (MI), and revascularization, along with the individual components of MACE, between the two groups during a long-term follow-up. **Results**: A total of 11,779 NSTEMI patients were included, with 4522 admitted to the ICCU and 7257 to the department of internal medicine. Patients admitted to the ICCU had lower systolic blood pressure, higher troponin levels and lower left ventricular ejection fraction (LVEF) compared to those in the IMD group, indicating greater initial clinical severity. Although patients admitted to the ICCU experienced a significantly higher rate of in-hospital complications, there were no significant differences in the incidence of in-hospital deaths between the two groups. During 5-year follow-up, NSTEMI patients initially admitted to the ICCU had significantly lower rates of mortality and MACEs. The estimated hazard ratio for 5-year MACE and 5-year mortality rates for NSTEMI patients admitted to the IMD vs. those admitted to the ICCU were 2.03 (95% CI, 1.04–3.34) and 2.5 (95% CI, 1.10–4.38), respectively. **Conclusions**: NSTEMI patients admitted to the ICCU experienced lower long-term mortality and MACE rates. These findings support the management of NSTEMI patients in cardiac wards and warrant further research into the reasons for the improved outcome.

## 1. Background

Patients with non-ST elevation myocardial infarction (NSTEMI) can be categorized into heterogeneous risk groups, among which the high-risk group’s mortality rates are similar to those of patients with ST elevation myocardial infarction (STEMI) [1]. Accordingly, current guidelines recommend the management of NSTEMI in intensive cardiac care units (ICCUs) equipped with monitoring capabilities and qualified staff [2]. Following admission and stabilization, along with medical treatment and hemodynamic monitoring, patients undergo coronary angiography and subsequent invasive treatment, which may include percutaneous coronary intervention (PCI) or coronary artery bypass graft (CABG) surgery. The timing of the intervention is guided by risk stratification recommendations [2,3]. ICCU is staffed with expert dedicated nurses and physicians equipped with appropriate advanced diagnostic facilities, essential for guiding the delivery of pharmacological and invasive treatment. Initial stratification and management of these patients, along with further long-term, guideline-directed therapy, are important prognostic factors.

However, due to logistical constraints, in many hospitals, NSTEMI patients are often admitted to general internal medicine departments (IMDs) rather than cardiac care units [4]. Data on the impact of admission wards on treatment and subsequent clinical outcomes of NSTEMI patients are limited.

Our study aims to evaluate the short- and long-term impact of the hospital admission ward on the clinical outcomes of NSTEMI patients, in a large tertiary medical center registry.

## 2. Methods

We conducted an observational retrospective study at Soroka University Medical Center, a large tertiary center in the south of Israel, which is the only medical center within a 100 km radius, providing care for approximately 1.2 million citizens [5,6]. We included consecutive patients admitted between January 2008 and December 2018, meeting the following criteria: age over 18 years, discharge diagnosis of NSTEMI based on the fourth universal definition of myocardial infarction (MI) [7].

Clinical and demographic data, including prior history, vital signs, medical treatment upon admission, laboratory tests, angiographic and imaging findings, and procedural data, were extracted from electronic medical records. The diagnosis of NSTEMI was based on clinical, electrocardiographic, and biochemical criteria, in accordance with the fourth universal definition of MI [7]. Patients were managed at the discretion of each admission ward.

Patients were divided into two groups according to the admission ward: IMD and ICCU. The assignment to these wards was determined by the attending physician based on clinical judgment, considering factors such as the patient’s severity of illness, the need for intensive monitoring, and the availability of specialized resources.

We compared baseline characteristics, treatment, and clinical outcomes between the two groups, in the short and long term of more than 5 years.

Clinical outcomes included major adverse cardiovascular events (MACEs)—a composite of all-cause death, recurrent MI, and unplanned ischemia-driven revascularization along with its individual components. The classification of recurrent MI was based on the fourth universal definition of MI [7]. Unplanned ischemia-driven revascularization was defined as revascularization because of angina symptoms, new ischemic changes on electrocardiography (ECG), or signs of reversible myocardial ischemia on non-invasive imaging.

Survival status was determined from hospital charts and by matching the identification numbers of the patients with the Israeli National Population Registry. This study was approved by the local institutional ethics committee of Soroka University Medical Center, in accordance with the principles of the Declaration of Helsinki.

## 3. Statistical Analysis

The study population’s characteristics were presented as *n* (%) for categorical variables and as median [interquartile range (IQR)] or mean (±sd) for normal/non-normal distributed continuous variables. Troponin measurements were expressed as times upper limit normal (ULN). The Chi-square test was used for categorical variables, and *t*-test or Mann–Whitney–Wilcoxon test was used as appropriate for continuous variables. Clinical outcomes are presented as Kaplan–Meier curves, and the log-rank test was used to test the differences in outcomes between admission wards.

To address baseline differences between groups and minimize confounding, we employed a double-robustness approach that combined inverse probability of treatment weighting (IPTW) with multivariable Cox proportional hazards regression. IPTW was used to balance covariates across the IMD and ICCU groups based on propensity scores, accounting for differences in patient characteristics, including age, gender, social score, body mass index (BMI), smoking, dyslipidemia, hypertension, diabetes mellitus (DM), atrial fibrillation, peripheral vascular disease (PVD), end-stage renal disease (ESRD), prior MI, prior PCI or CABG, and clinical and laboratory parameters on admission (e.g., blood pressure, heart rate, creatinine, hemoglobin, white blood cell count, troponin, left ventricular ejection fraction (LVEF), multivessel disease, vascular access, and time from admission to PCI). The Cox regression model was subsequently applied to adjust for any residual imbalance not addressed by IPTW, enhancing the validity of the results.

Statistical significance was considered at *p* < 0.05 in univariable tests. Missing values in the included covariates were less than 10% and were not imputed. All analyses were performed using R (V.4.0.3, R Foundation for Statistical Computing, Vienna, Austria).

## 4. Results

A total of 11,779 NSTEMI patients were included in this study, of whom 4522 (38.4%) were admitted to ICCU and 7257 (62.6%) to IMD. The baseline characteristics according to admission wards are presented in Table 1. ICCU patients were younger (mean age of 62.7 y vs. 67.6 y, *p* < 0.001), more frequently males (78.1% vs. 68.1%, *p* < 0.001) and smokers (56% vs. 45%, *p* < 0.001); they were less frequently hypertensive (55.2% vs. 65.7%, *p* < 0.001) and diabetic (39.2% vs. 48.6%, *p* < 0.001). Only a minority of patients in the two groups had experienced a prior MI (14.7% and 18.9% in the ICCU and IMD group, respectively).

The clinical characteristics of the study population upon hospital admission according to admission wards are presented in Table 1. In the ICCU group, patients presented with significantly lower systolic blood pressure (SBP) (mean 127 vs. 135 mmHg, *p* < 0.001), with a higher rate of patients having SBP below 90 mmHg (3.2% vs. 1.3%, *p* < 0.001). The maximal troponin measured was higher in the ICCU group (7.1 vs. 5.59 times ULN, *p* < 0.001). Moreover, ICCU patients were more likely to have LVEF below 35% (18.6% vs. 16.9%, *p* = 0.024).

Differences in in-hospital interventional treatment and procedural data are presented in Table 2. Patients admitted to the ICCU more frequently underwent invasive coronary angiography (ICA) within 72 h of admission (26% vs. 5% underwent ICA within 24 h and 51.6% vs. 22.2% within 24–72 h, *p* < 0.001), whereas the majority of patients admitted to the IMD underwent ICA more than 72 h from admission (72.8%). More patients in the ICCU group were referred to coronary artery bypass graft (CABG) to complete revascularization (14% vs. 11.9%, *p* = 0.002).

**Figure 1:**
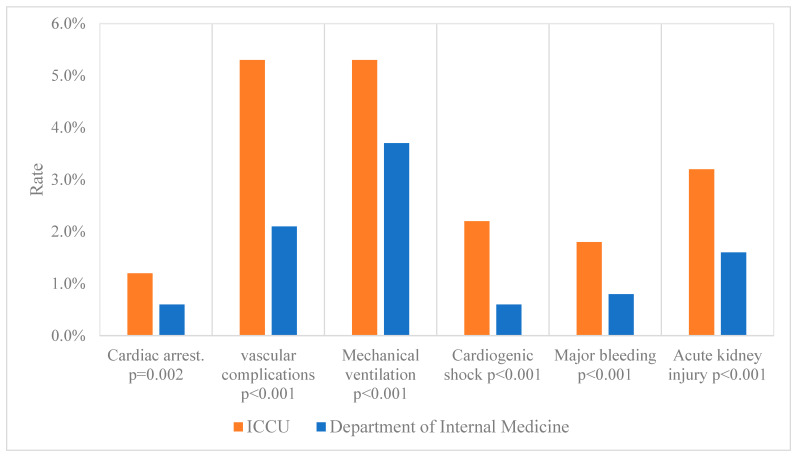
In-hospital post-percutaneous coronary intervention complications according to admission wards. In-hospital major complications rates, including cardiac arrest, vascular complications, mechanical ventilation, cardiogenic shock, major bleeding, and acute kidney injury in the department of internal medicine group and intensive cardiac care unit group. Values are presented as mean (percentage).

In-hospital complications are presented in Figure 1. Although patients admitted to the ICCU experienced significantly more complications, including cardiac arrest, respiratory deterioration with the need for mechanical ventilation, cardiogenic shoch, major bleeding, and acute kidney injury, there were no significant differences in the incidence of in-hospital death between the two groups. The length of hospital stay was significantly shorter in the ICCU group (median 5 days [IQR, 4 to 8] vs. 8 days [IQR, 5 to 14], *p* < 0.001).

**Figure 2:**
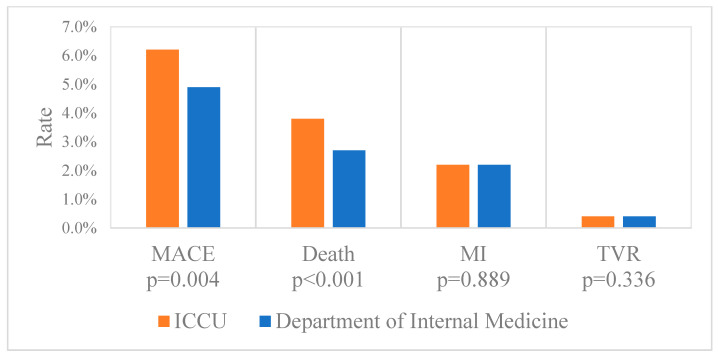
Thirty-day clinical outcomes according to admission wards. Thirty-day clinical outcome rates, including MACE, all-cause death, MI, and TVR, in the department of internal medicine group and intensive cardiac care unit group. Values are presented as mean (percentage). MACE = major adverse cardiovascular events; MI = myocardial infarction; TVR = target vessel revascularization.

Thirty-day mortality and MACE occurred more frequently in the ICCU group (6.2% vs. 4.6%, *p* = 0.004 and 3.8% vs. 2.7%, respectively, *p* < 0.001), while no significant difference was noted in 30-day recurrent MI or target vessel revascularization (TVR) (Figure 2).

**Figure 3:**
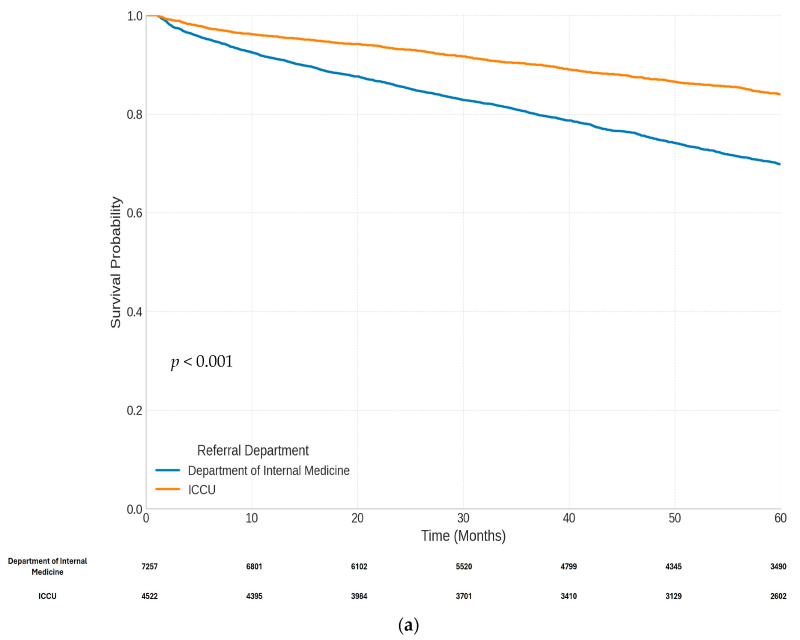

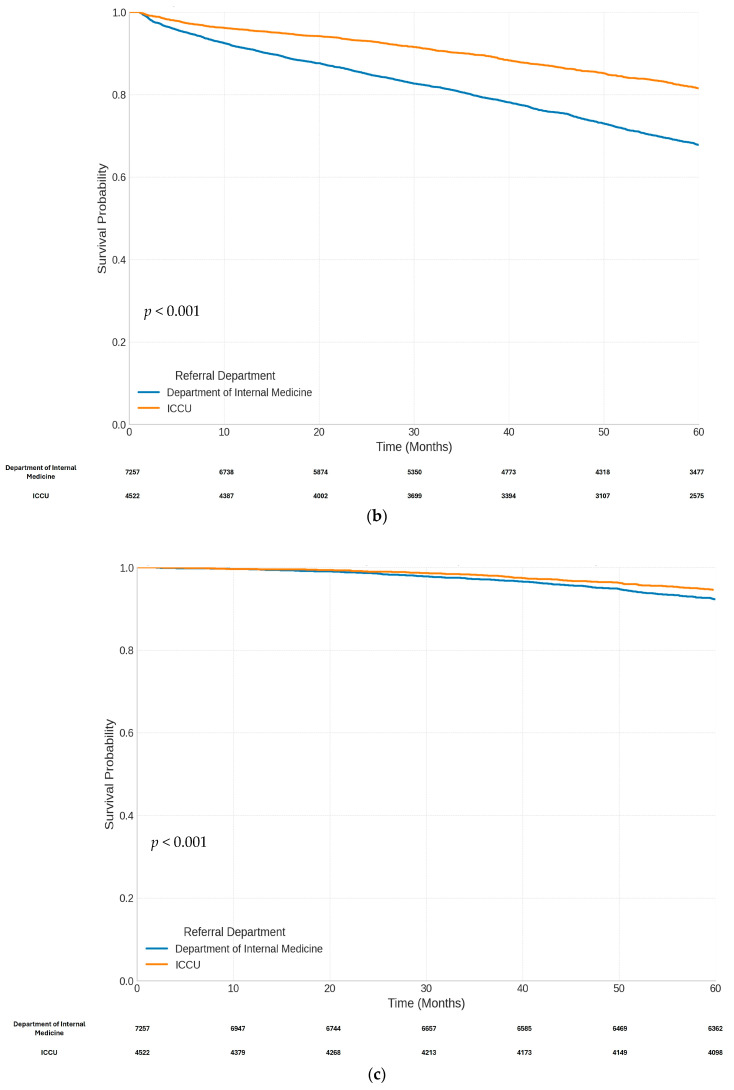
Kaplan–Meier curves for 5-year outcomes according to the study groups. (**a**) Kaplan–Meier curve of 5-year mortality in patients in the department of internal medicine and intensive cardiac care unit groups. (**b**) Kaplan–Meier curve of 5-year MACE in patients in the department of internal medicine and intensive cardiac care unit groups. (**c**) Kaplan–Meier curve of 5-year recurrent MI in patients in the department of internal medicine and intensive cardiac care unit groups.

The long-term follow-up results, of over 5 years (median 64 months [IQR, 39 to 95]), are presented in Figure 3. NSTEMI patients admitted to ICCU had significantly lower 5-year mortality (Figure 3a) and 5-year MACE (Figure 3b). The overall rate of recurrent MI at the 5-year follow up was low and occurred less frequently in the ICCU group (Figure 3c). Low rates of unplanned revascularization were observed in the two groups.

**Figure 4:**
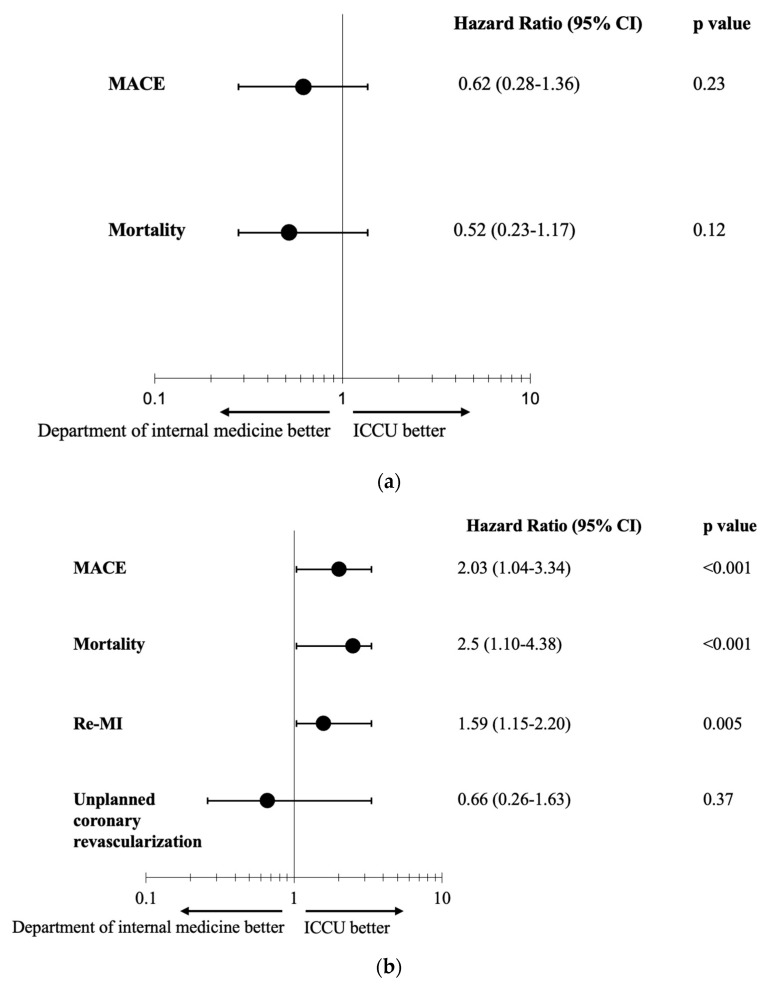
The association between hospitalization in intensive cardiac care unit or internal medicine department and cardiovascular morbidity and mortality outcomes: 30-day follow-up and 5-year follow-up. Results of adjusted propensity score model: (**a**) 30–day follow-up; (**b**) 5–year follow-up (1–60 months). CI = Confidence Interval; MACE = major adverse cardiovascular events; MI = myocardial Infarction.

The inverse propensity-weighted cox proportional hazard model is presented in Figure 4 and shows the association between admission ward and clinical outcomes. Among NSTEMI patients who were admitted to IMD, the estimated hazard ratios (HRs) for 30-day MACE and 30-day mortality rates were 0.62 (95%CI, 0.28 to 1.36, *p* = 0.23) and 0.52 (95%CI, 0.23 to 1.17, *p* = 0.12), respectively (Figure 4a).

During the longer-term follow-up of 5 years, the estimated HRs for 5-year MACE and 5-year mortality rates for NSTEMI patients who were admitted to IMD were 2.03 (95%CI, 1.04 to 3.34, *p* < 0.001) and 2.5 (95%CI, 1.10 to 4.38, *p* < 0.001), respectively (Figure 4b). Moreover, Figure 4b shows that the estimated HR for 5-year re-MI for patients admitted to IMD was 1.59 (95%CI, 1.15 to 2.2, *p* = 0.005). There was no significant association between admission ward and revascularization in the 5-year follow-up.

## 5. Discussion

Our study aimed to compare MACE, including all-cause death, recurrent MI, and revascularization, along with individual components of MACE, between patients with NSTEMI admitted to either the ICCU or the IMD at a large university tertiary center. This study followed patients for up to 5 years. Our results indicate that despite the differences in baseline characteristics and clinical presentation, outcomes for patients initially admitted to the ICCU appeared worse than among those admitted to IMD at short-term follow-up but improved significantly over the long term, including lower rates of mortality, re-MI, and MACE, at 5-year follow-up. The worse short-term clinical outcomes in the ICCU group could be related to higher risk parameters on admission and a higher rate of complications during hospitalization. Nonetheless, as shown in the adjusted propensity score model, there was no significant association between admission ward and 30-day mortality and MACE rates.

Compared with patients admitted to IMD, those admitted to the ICCU tended to be younger and male. This observation is consistent with worldwide trends [8,9]. Patients admitted to the ICCU typically presented with lower systolic blood pressure, higher troponin levels, and lower LVEF, which are indicative of increased myocardial injury and higher initial risk [10,11,12], which could explain the higher short-term mortality. Management by cardiac staff, coupled with advanced invasive monitoring technologies and higher nurse-to-patient ratios, might have contributed to more effective management of complications [13,14]. As noted, there was a lack of significant differences in in-hospital mortality between the two groups, despite higher complication rates in the ICCU, highlighting the effectiveness of specialized cardiac care.

The impact of admission wards on the management and outcomes of NSTEMI patients has been the subject of several studies [15,16,17]. Most of these studies primarily evaluated the impact of the specialty of admitting physicians, often comparing cardiologists to general medical care physicians, rather than examining the type of ward to which patients are admitted. This focus introduces a limitation in those studies, as they do not isolate the independent association between the ward of admission and patient outcomes. In contrast, our study specifically investigates the independent role of admission ward type (ICCU vs. IMD) on long-term clinical outcomes, which provides more direct insight into the effects of ward-based management.

Additionally, a recently published national registry study by Moledina et al. [14] highlighted differences in the care and management of 300,000 NSTEMI patients admitted to cardiac and non-cardiac wards. Consistent with our findings, patients admitted to cardiac wards were younger, predominantly male, and more likely to undergo ICA and PCI within 24 h of admission. However, the study by Moledina et al. focused primarily on in-hospital mortality and MACE, without extending its analysis to long-term outcomes. Our study extends the existing literature by providing comprehensive data on long-term follow-up, spanning over 5 years, which allows for a deeper understanding of how early interventions and the type of ward influence long-term outcomes in NSTEMI patients.

Moreover, the Moledina et al. study involved multiple medical centers with varying structural characteristics and standards of care, which may limit the generalizability of their results to specific healthcare settings. In contrast, our study was conducted at a single tertiary medical center, offering a more controlled setting to assess the impact of ward type on long-term patient outcomes.

A key observation was the significant difference in the timing of PCI between the two groups. Patients in the ICCU were more likely to receive PCI within the recommended 24–72 h, while delayed intervention (>72 h) was more common in the IMD group. The current NSTEMI guidelines strongly emphasize early invasive strategies for high-risk patients, as delayed revascularization has been associated with poorer outcomes [18,19]. Our study underscores the importance of timely interventions, particularly in ICCU settings, where adherence to these guidelines was more consistent. However, the improved long-term outcomes observed in the ICCU group cannot solely be attributed to early invasive management.

Our enhanced long-term clinical outcome findings for ICCU patients may also be explained by a better pharmacological-guided, directed treatment, medical adherence and persistence education, higher referral to cardiologist specialties’ ambulatory follow-up, cardiac rehabilitation programs, and professional lifestyle modification advice for secondary prevention given to patients by expert staff in the ICCU prior to discharge [2]. This comprehensive approach, including both acute management and rigorous post-discharge follow-up, likely contributed to the observed improvement in long-term outcomes.

In the study period, only one in four NSTEMI patients were admitted to the ICCU, and our study implies that changing this practical management of patients presenting with NSTEMI may improve their long-term outcomes.

There are several important limitations in our study that should be taken into consideration. First, it is an observational single-center study. Patients’ allocation to ICCU or IMD was determined by the attending physician according to perceived patient risk and bed availability. Therefore, unmeasured confounding differences may exist between the two groups despite our efforts for adjustment. Furthermore, practices vary between centers, which may limit the generalizability of our findings. Second, we were not able to calculate an accepted risk score, such as GRACE score [20], for the entire study population. Third, our data regarding re-MI and unplanned ischemia-driven revascularization included patients who were re-admitted only to our medical center, with no information available about admission or re-interventional treatment in other centers; however, Soroka Medical Center is the only hospital within a 100 km radius [5,6]. Finally, our data lack information on secondary prevention medications prescribed at discharge in the study groups, which may affect the assessment of post-discharge treatment adherence and outcomes.

## 6. Conclusions

In conclusion, our study demonstrated significantly lower rates of long-term mortality, MI, and MACE among NSTEMI patients managed in wards with specialized cardiac expertise. These findings provide further support for the management of NSTEMI patients in cardiac wards to improve long-term clinical and survival outcomes. Further research is warranted in order to elucidate the precise reasons for improved clinical outcomes.

## Figures and Tables

**Table 1:** Baseline demographic and clinical parameters.

	Department of Internal Medicine	ICCU	*p* Value
*n*	7257	4522	
Age, years (mean) [±SD]	67.6 (±12.1)	62.7 (±12.9)	<0.001
Gender (male)	5013 (69.1%)	3532 (78.1%)	<0.001
BMI (kg/m^2^) (mean) [±SD]	29.3 (±9.40)	28.7 (±9.16)	<0.001
Current smokers	3265 (45.0%)	2533 (56.0%)	<0.001
Dyslipidemia	5622 (77.5%)	3538 (78.2%)	0.34
Hypertension	4767 (65.7%)	2495 (55.2%)	<0.001
Diabetes mellitus	3525 (48.6%)	1772 (39.2%)	<0.001
HBA1C (%) (mean) [±SD]	7.04 (±1.85)	6.68 (±1.73)	<0.001
Atrial fibrillation	1888 (26.0%)	961 (21.3%)	<0.001
PVD	625 (8.6%)	307 (6.8%)	<0.001
Dialysis	261 (3.6%)	110 (2.4%)	<0.001
COPD	204 (2.8%)	72 (1.6%)	<0.001
Prior to admission			
MI	1374 (18.9%)	666 (14.7%)	<0.001
CABG	1094 (15.1%)	485 (10.7%)	<0.001
PCI	2663 (36.7%)	1322 (29.2%)	<0.001
Characteristics on admission			
Pulse (bpm)	71.3 (±14.9)	72.0 (±13.8)	<0.001
SBP (mmHg)	135 (±23.7)	127 (±22.8)	<0.001
SBP < 90 (mmHg)	94 (1.3%)	145 (3.2%)	<0.001
Creatinine (mg/dL)	1.28 (±1.34)	1.11 (±1.04)	<0.001
Hemoglobin (g/dL)	12.8 (±2.03)	13.4 (±2.00)	<0.001
WBC (10^3^/uL)	8.90 (±3.94)	10.2 (±4.27)	<0.001
Troponin (max/ULN)	5.59 (±4.55)	7.10 (±4.92)	<0.001
Reduced LV function	1230 (16.9%)	841 (18.6%)	0.024
LVEF (%)	56.1 (±16.3)	56.7 (±15.2)	0.608

Data are presented as mean (±SD), or number (percentage). BMI = body mass index; CABG = coronary artery bypass graft; COPD = chronic obstructive pulmonary disease; LV = left ventricle; LVEF = left ventricular ejection fraction; MI = myocardial infarction; PCI = percutaneous coronary intervention; PVD = peripheral vascular disease; SBP = systolic blood pressure; WBC = white blood cells.

**Table 2:** In-hospital interventional treatment and procedural data.

	Department of Internal Medicine	ICCU	*p* Value
*n*	7257	4522	
Interventional treatment characteristics			
Time To PCI (hours)			<0.001
<24	363 (5.0%)	1175 (26.0%)	
24–72	1610 (22.2%)	2333 (51.6%)	
>72	5284 (72.8%)	1014 (22.4%)	
Therapeutic PCI	6391 (88.1%)	3891 (86.0%)	0.002
CABG	866 (11.9%)	631 (14.0%)	0.002
Thrombolytic therapy	81 (1.1%)	298 (6.6%)	<0.001
Procedural findings and modalities			
Access site			<0.001
Femoral	1598 (22.0%)	830 (18.4%)	
Radial	5480 (75.5%)	3571 (79.0%)	
Other	179 (2.5%)	121 (2.7%)	
LM stenosis > 50%	892 (12.3%)	507 (11.2%)	0.083
Multi vessels disease	3000 (41.3%)	1897 (42.0%)	0.525

Data are presented as mean (SD), or number (percentage). CABG = coronary artery bypass graft; LM = left main artery; PCI = percutaneous coronary intervention.

## Data Availability

The original contributions presented in this study are included in the article. Further inquiries can be directed to the corresponding author(s).
